# Initial Evidence for the Hypersensitivity Hypothesis: Emotional Intelligence as a Magnifier of Emotional Experience

**DOI:** 10.3390/jintelligence9020024

**Published:** 2021-05-04

**Authors:** Marina Fiori, Andrew Ortony

**Affiliations:** 1 Swiss Federal Institute for Vocational Education and Training (SFIVET), 1020 Lausanne, Switzerland; 2 Department of Psychology, Northwestern University, Evanston, IL 60208, USA; ortony@northwestern.edu

**Keywords:** hypersensitivity, emotional intelligence, ability EI, social perception, amplification of emotions, emotion understanding

## Abstract

In this article, we provide preliminary evidence for the ‘hypersensitivity hypothesis’, according to which Emotional Intelligence (EI) functions as a magnifier of emotional experience, enhancing the effect of emotion and emotion information on thinking and social perception. Measuring ability EI, and in particular Emotion Understanding, we describe an experiment designed to determine whether, relative to those low in EI, individuals high in EI were more affected by the valence of a scenario describing a target when making an affective social judgment. Employing a sample of individuals from the general population, high EI participants were found to provide more extreme (positive or negative) impressions of the target as a function of the scenario valence: positive information about the target increased high EI participants’ positive impressions more than it increased low EI participants’ impressions, and negative information increased their negative impressions more. In addition, EI affected the amount of recalled information and this led high EI individuals to intensify their affective ratings of the target. These initial results show that individuals high on EI may be particularly sensitive to emotions and emotion information, and they suggest that this hypersensitivity might account for both the beneficial and detrimental effects of EI documented in the literature. Implications are discussed.

## 1. Introduction

Emotional Intelligence (EI) was introduced into the psychological literature as a form of intelligence that concerns using emotions to guide thinking and action (Salovey and Mayer 1990). Since its introduction, there has been much discussion as to how best to characterize and measure the construct, and as to whether it really is a distinct construct separate from personality and intelligence (see, e.g., Matthews et al. 2004). Two conceptually different approaches have been developed to study EI: the trait approach in which the construct is referred to as *trait EI*, and the ability approach, *ability EI* (Petrides and Furnham 2001). The first views EI as a disposition and uses self-report questionnaires, such as the Trait Emotional Intelligence Questionnaire (TEIQue, Petrides 2009) to measure the construct; the second conceptualizes EI as the ability to process emotion information and is measured with performance tests, such as the Situational Test of Emotion Understanding (STEU, MacCann and Roberts 2012). Although the two approaches broadly refer to the same construct, there are important differences between them.

Perhaps because of the urgency of addressing the general issue of the validity and measurement of the EI construct, scholars have devoted relatively little attention to understanding the cognitive processes underlying individual differences in EI (Fiori 2009; Gutiérrez-Cobo et al. 2016; Mestre et al. 2016). Whereas we know that EI is associated with (mostly) positive outcomes in domains such as health (Austin et al. 2005), teachers’ well-being (Vesely et al. 2014), interpersonal effectiveness (Fiori 2015), job performance (Joseph and Newman 2010), and academic achievement (MacCann et al. 2020), we do not know what type of emotional and cognitive processes account for such outcomes and how they operate in high as compared to low EI individuals (Fiori 2009). Understanding the cognitive processes through which EI exerts its effects is likely to contribute to a better conceptualization of EI and to a better understanding of how EI may lead to the outcomes documented in the literature. To start our discussion of relevant previous research, we first consider some ways in which individuals vary in processing information as a function of their level of EI, where necessary, specifying whether the effects that we describe were associated with trait EI or ability EI.

In a review of the literature on the relationship between EI and cognitive processes, Gutiérrez-Cobo et al. (2016) report that research using perceptual, attentional, and other kinds of cognitive tasks reveals that high EI individuals tend to process emotion information more efficiently than low EI individuals. For example, high EI individuals tend to be more accurate and faster at recognizing and paying attention to emotional stimuli, especially when assessed using measures of ability EI as opposed to trait EI. At the same time, there appears to be no systematic relationship between cognitive tasks involving non-emotional stimuli and EI, regardless of the type of measure employed. In their conclusion, the authors acknowledge a need for a more fine-grained analysis of the cognitive processes associated with EI, as well as for the use of research designs that would allow for the testing of the causal role of such processes on EI-related outcomes. 

Some scholars have proposed examining the mechanisms underlying EI by appealing to the appraisal processes associated with emotional experience. These processes, they believe, might explain more socially effective and personally beneficial consequences of EI (Fontaine 2016; Mestre et al. 2016; Roberts et al. 2016). An approach to mechanisms underlying EI, discussed in another review of the literature (Lea et al. 2019), explores the possibility that EI might work as a “stress buffer” that lowers acute stress in emotionally demanding situations, a view initially proposed by Mikolajczak et al. (2009). Their results showed that the association between EI and stress reactivity and recovery differed depending on the stress context and on how stress was measured, with trait EI helping only in some contexts. Furthermore, and of particular interest in the present context, Lea and colleagues reported that in certain situations, ability EI showed a deleterious effect on stress reactions and recovery. 

This last-mentioned finding suggests that even though EI is generally considered to be beneficial, it may not always lead to positive outcomes. And, in fact, despite the burgeoning literature showing the positive effects of EI, the idea that EI might sometimes have deleterious side effects has been suggested in the mental health domain (Davis and Nichols 2016), as well as being reminiscent of a debate in industrial-organizational psychology about whether people in leadership roles need emotional intelligence. Central to that debate was the question of whether high EI individuals might be overly affected by emotions felt by themselves and by others in a way that would hamper their effectiveness as leaders in the workplace. This effect was called the “curse of emotion”, the idea being that high EI leaders might be insufficiently assertive when having to deal with controversial issues, thus compromising their ability to function effectively (Antonakis et al. 2009). A preliminary test of the curse of emotion idea was provided in a study in which high EI individuals were more strongly affected by incidental anger and provided more biased ratings of the characteristics of an ambiguous target (Fiori and Ortony 2016). The same study also introduced a potential explanation of this finding––an explanation that we dubbed the “hypersensitivity hypothesis” whereby individuals high in EI may be more sensitive to emotions and to emotion information than individuals low in EI. 

We believe that an interesting perspective regarding possible outcomes of EI, including beneficial or adverse consequences for an individual, can be revealed by considering a different function of EI, namely, its function as a *magnifier of emotional experience,* meaning that (high) EI amplifies the valenced aspects of experience. According to this view, individuals high in EI are emotionally hypersensitive in that they experience stronger emotions and pay more attention to their own and others’ emotions. In other words, relative to individuals not high in EI, their positive experiences are more positive, and their negative experiences more negative, and this amplification of affect can lead to changes in behavior and social perception. If EI functions as a magnifier in this way, one might suppose that it could have both advantageous and adverse *consequences.* For example, in some cases having a deeper, more fine-grained understanding of the emotional reactions of themselves and others could provide additional information that would help better navigate the social environment, thus retaining the benefits of high EI. However, in other cases, the additional emotion information conferred by the magnifying effects of high EI might overtax cognitive resources and lead to suboptimal decisions and actions, consistent with the kind of detrimental effects discussed above.

We now review some instances in which high EI has been shown to have negative consequences. To date, most of the empirical work addressing the detrimental effects of EI concerns how EI relates to coping with stress. A study by Bechtoldt and Schneider (2016) found that men with higher basal testosterone and who were also high in the emotion perception component of ability EI showed higher cortisol levels during a situation of social stress. Similarly, Matthews et al. (2006) reported that high EI individuals—again with EI measured as an ability—showed a more relaxed mood before a stress induction but more distress after executing a stressful task. Using a measure of trait EI, Mikolajczak et al. (2009) found that individuals high in trait EI, in particular in self-control, recalled more negatively valenced information than positive and neutral information when exposed to a stressful condition, and Davis and Humphrey (2012) found that that although trait EI contributed to attenuating the relationship between stressors and mental health, ability EI amplified the relationship between economic deprivation and depression. 

Research of the kind just mentioned reports detrimental effects of EI at the intrapersonal level, in contrast to work that addresses detrimental effects at the interpersonal level (Schlegel 2020). With reference to the latter, Baker et al. (2013) found that greater trait EI is associated with more sympathetic responses to deceptive as opposed to truthful individuals. These authors suggested that high EI individuals might have given more weight to emotional cues, which are generally more pronounced in deceptive individuals, which then triggered stronger sympathetic responses and led high EI individuals to believe that deceivers were in fact sincere. Overall, their results suggest that stronger receptivity to emotional signals hinders accurate judgments. 

The above discussion suggests that both ability and trait EI are involved in potential downsides of EI, and that of the four branches of the standard Salovey and Mayer (1997) ability model of EI (Perceiving, Using, Understanding, and Managing Emotions), the one facet that seems to be particularly involved whenever non-advantageous effects of EI are observed is that of emotion perception (see also Schlegel 2020). However, the studies conducted to date are too few and too fragmented to enable any compelling conclusions to be drawn regarding whether an amplification effect of EI might involve only some branches of EI or all of them. 

Finally, there is no *a priori* reason to believe that any effect of high EI on the processing of emotion and emotion information is limited to the amplification of negative information. A study using eye-tracking showed that individuals high in trait EI paid more attention to happy than to negative and neutral faces (Lea et al. 2019), which, incidentally, is in sharp contrast to the standard “pop-out” advantage of angry over happy faces (Hansen and Hansen 1988). In general, EI is known to be associated with positive mood: high EI individuals reacted with higher positive mood after a positive emotion induction and a lower decrease of positive mood after a negative emotion induction (Schutte et al. 2001). This kind of amplification of positive emotion has also been confirmed by other studies (e.g., Fernández-Berrocal and Extremera 2006; Petrides and Furnham 2001). However, these same studies also revealed that high self-reported EI individuals sometimes amplify negative emotions, experiencing stronger negative feelings, in particular anger, after a negative emotion induction. 

Thus, in sum, there is evidence that regardless of whether measured using trait EI or ability EI, high EI, although often conferring advantages to individuals, can sometimes result in outcomes that can be disadvantageous to the individual, and there is also reason to believe that one function of EI might be to amplify the effects of valenced information on emotional experience.

### 1.1. The Current Study

We here report a study designed to test more directly the hypothesis that (ability) EI functions as a magnifier of emotional information. We examined two types of cognitive processes that might play a role in such a magnifying effect, namely the *perception* of emotional content, and the *retrieval* of such information. If the ability to quickly and efficiently determine whether and to what extent presented information is emotional in nature constitutes a foundational ability of EI, then high EI individuals should outperform low EI individuals on a task such as story understanding that involves the processing of emotion-related information. In addition, and as a consequence of their deeper processing of emotional information, high EI individuals should show better memory of emotional content. Because our hypothesis of EI as a magnifier applies to both positive and negative information, the current study employed both positive, negative content, along with neutral content. 

For several reasons, we focused on the *Emotion Understanding* component of the Salovey and Mayer (1997) ability model: First, from a conceptual point of view the relationship between the type of cognitive processes involved in the current study, namely perception and retrieval of emotion information conveyed through language, should be stronger for EI conceptualized as the capacity to process and use emotion information to support thinking and behavior (Mayer et al. 2008). Second, emotion understanding is the branch most strongly involved in the perception and understanding of (verbally conveyed) emotional content. Third, this EI component has the strongest factor loading on ability EI (MacCann et al. 2014). 

#### Hypotheses

We manipulated the content of a vignette describing an individual called Donald so as to vary the valence of the information provided, making it either positive, negative, or neutral (see Appendix A). Based on our view of EI as a magnifier of emotional experience, we hypothesized that ratings of impressions about Donald would be influenced by the overall affective tone of the vignette, but more so for high than for low Emotion Understanding participants. Specifically, we expected that individuals high in the Emotion Understanding component of ability EI would:

**Hypothesis** **1.** *When exposed to a predominantly positive vignette, report more positive impressions than those low in Emotion Understanding.*

**Hypothesis** **2.** *When exposed to a predominantly negative vignette, report more negative impressions than those low in Emotion Understanding.*

We did not have specific hypotheses regarding differences between high and low Emotion Understanding in the neutral vignette condition, although we thought that in the neutral condition difference between high and low Emotion Understanding might disappear. 

**Figure 1:**
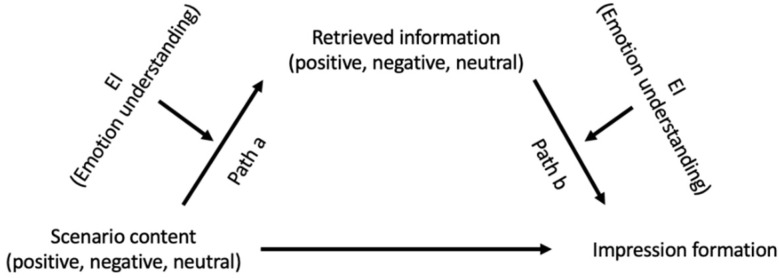
Illustration of Hypothesis 3 and 4 (moderated mediation).

We also hypothesized that the effect of Emotion Understanding on the relationship between the content of the vignette and ratings of Donald would be mediated by the type of retrieved information as in a moderated mediation model (Figure 1). In particular, we expected that:

**Hypothesis** **3.** *Retrieval of negative emotion information would mediate the relationship between negative scenario and impression formation for participants high in Emotion Understanding.*

**Hypothesis** **4.** *Retrieval of positive emotion information would mediate the relationship between positive scenario and impression formation.*

We measured the general mood of participants at the beginning of the experimental session and ability EI using the Situational Test of Emotion Understanding (STEU; MacCann and Roberts 2008).

### 1.2. Participants and Procedure

Participants were one hundred and sixty-five recruited through the online platform Mechanical Turk. They were remunerated for their participation. We employed a strict procedure to select participants for the study, and excluded the data of 35 participants either because they failed to fill out the entire initial mood questionnaire (25), or because they failed to recall details of the scenario (20). Of the final sample of 130 participants, 71% percent were male and 26% were female; 3% did not indicate their gender. The employment status was 27.9% employed full-time, 22.4% employed part-time, 23.6% students, and 5.5% unemployed. Mean age was 28.79 (*SD* = 9.36), with age range between 18 and 66. Participants first completed a current mood questionnaire, and then were randomly assigned to read one of three scenarios (positive, negative, or neutral), after which they rated the character described in the vignette (Donald) on 12 traits, 6 of which were positive and 6 negative. They were then asked to recall at least five details of the scenario they had read. The ability EI test (STEU) was completed at the end so that the processing of its items (which involved reading about emotion situations) would not influence participants’ interpretations of the stimulus materials. The entire procedure took an average of about 25 min to complete. The study received ethical approval from the local University IRB. 

### 1.3. Design

The study was a mixed design with Emotion Understanding and retrieval of information measured across participants, and scenario content (positive, negative, neutral) manipulated between participants. The dependent variable was impression formation ratings, which were calculated as the average ratings across the 12 adjectives used to rate the target individual.

### 1.4. Measures

#### 1.4.1. Brief Mood Introspection Scale (BMIS)

To check for participants’ initial mood, we employed the BMIS (Mayer and Gaschke 1988), which includes a list of 16 adjectives applicable to emotional states, each with a 4-point likert scale to indicate the strength of the feeling, followed by an overall evaluation of current mood, ranging from 1 (very unpleasant) to 20 (very pleasant). This instrument was administered in order to check the initial emotional state of participants before they read the scenario and rated the target person, a purpose best served by using the one-item overall mood.

#### 1.4.2. Scenario Content

We modified the vignette employed in Srull and Wyer (1979) in which a narrator describes an afternoon spent with an acquaintance, Donald. In the original Srull and Wyer study, five somewhat hostile but ambiguous behaviors (e.g., demanding money back from a salesclerk) were embedded in the passage. In the present study we used three similar length versions (about 280 words), but varied their content so that in addition to 14 neutral idea units (Schiefele and Krapp 1996) the positive version had 7 positive idea units, the negative had 7 negative idea units, and the neutral version had 7 additional neutral idea units (see Appendix A for all three versions). Seven idea units were chosen because they represented one third of the total content and appeared enough to capture individual differences in the nuances of positivity and negativity of the vignette description, ensuring that the scenarios contained a certain level of ambiguity, which was helpful to capture variability in impression formation.

#### 1.4.3. Impression Formation

Each participant read one of the versions on the computer screen and then, using a unipolar 9-point likert scale, rated Donald on six negative traits (hostile, unfriendly, dislikable, boring, selfish, narrow-minded) and six positive traits (kind, considerate, thoughtful, intelligent, interesting, helpful). The scores on the positive trait descriptors were reverse coded and then all the ratings averaged across the 12 descriptors, with higher scores indicating more negative impressions, and lower scores more positive ones.

#### 1.4.4. Retrieved Information

Upon completing the impression formation task, participants were asked to recall at least five details of the scenario they had read. Their responses were scored for gist (as opposed to verbatim) recall. Recall content was coded by a researcher blind to the study hypotheses, who counted the number of positive, negative, and neutral idea units provided by each participant. 

#### 1.4.5. Emotion Understanding

EI was measured with the STEU (MacCann and Roberts 2012), which requires respondents to read 25 short scenarios and after each one to indicate how the protagonist described would feel. For example, one item read “Xavier completes a difficult task on time and under budget. Xavier is most likely to feel…?” and respondents have to select one of five emotions response choices. The MacCann and Roberts scoring protocol is based on Roseman’s (2001) appraisal theory of emotions. The Cronbach alpha reliability of the scale in the current sample was .84.

### 1.5. Data Analysis Plan

To test whether perceptions of the character (Donald) described in the vignettes varied as a function of the level of Emotion Understanding and the content of the vignette (Hypotheses 1 and 2), we conducted a linear regression in which we employed vignette content (positive, negative, and neutral) as a dummy coded categorical variable, Emotion Understanding (STEU score) as a continuous variable, and impression formation as the outcome variable.

To test whether the effect of vignette content (positive, negative, or neutral) on impression formation was mediated by the valence of retrieved information and moderated by Emotion Understanding (Hypotheses 3 and 4), we conducted two mediated moderation analyses, one for positive vignette content and retrieval of positive content, and the other for negative vignette content and negative retrieval of information. In all analyses, we controlled for overall mood, age, and sex.

## 2. Results

### 2.1. Descriptive Statistics

Mean, standard deviations and correlations are reported in Table 1.

On average participants, having been asked to recall at least five details, recalled 5.8 idea units (*SD* = 2.17). The correlation between the total amount of information recalled across all three conditions and the amount of positive information recalled was *r* = .16 (*p* = .07) and the correlation between total amount of information recalled and negative information recalled was *r* = .14 (*p* = .12). On the other hand, the total amount of information recalled was highly correlated with the amount of neutral information recalled, *r* = .68, *p* = .00, which is understandable given that all the scenarios shared the same 14 neutral action units.

Because the hypersensitivity hypothesis is a hypothesis about sensitivity to *emotional* information, we also tested whether any such differences would be evident in the neutral content condition. To check for the manipulation of the scenario content, we investigated whether the number of positive, negative, and neutral idea units recalled differed as a function of the three experimental conditions. Participants exposed to the positive scenario recalled more positive idea units (*M* = 1.72, *SD* = 1.79) than negative (*M* = .20, *SD* = .58) and neutral idea units (*M* = .19, *SD* = .50), *F* (2, 127) = 24.75, *p* < .001. Participants exposed to the negative scenario recalled more negative idea units (*M* = 2.61, *SD* = 1.94) than positive (*M* = .21, *SD* = .85) and neutral ones (*M* = .11, *SD* = .39), *F* (2, 127) = 53.56, *p* < .001, and participants exposed to the neutral scenario recalled more neutral idea units (*M* = 5.40, *SD* = 2.17) than negative (*M* = 3.0, *SD* = 2.25) and positive idea units (*M* = 4.0, *SD* = 2.57), *F* (2, 127) = 10.73, *p* < .001. There was no significant correlation between the score of Emotion Understanding and the number of positive and neutral idea units recalled, whereas there was a positive correlation between Emotion Understanding and the number of negative idea units recalled, *r* = .24, *p* < .01.

We first examined the correlation between the overall endorsements of positive and negative trait descriptors employed in the impression formation task. Ratings of positive descriptors were negatively and significantly correlated with ratings of negative descriptors (*r* = −.54), thus they were reverse coded and added to the negative adjective’s ratings, yielding an overall impression formation score in which higher values indicate more negative evaluations, and lower values more positive ones. 

### 2.2. Hypothesis Testing

To test the two parts of the hypersensitivity hypothesis (Hypotheses 1 and 2), a multiple regression was conducted using STATA version 14 (StataCorp 2019). The overall model was significant, *F* (8, 103) = 17.62, *p* < .001, *R*^2^ = .56. Among the focal predictors and the control variables, only Emotion Understanding showed a significant main effect, *B* = 2.96, *p* < .001 (Table 2).

**Figure 2:**
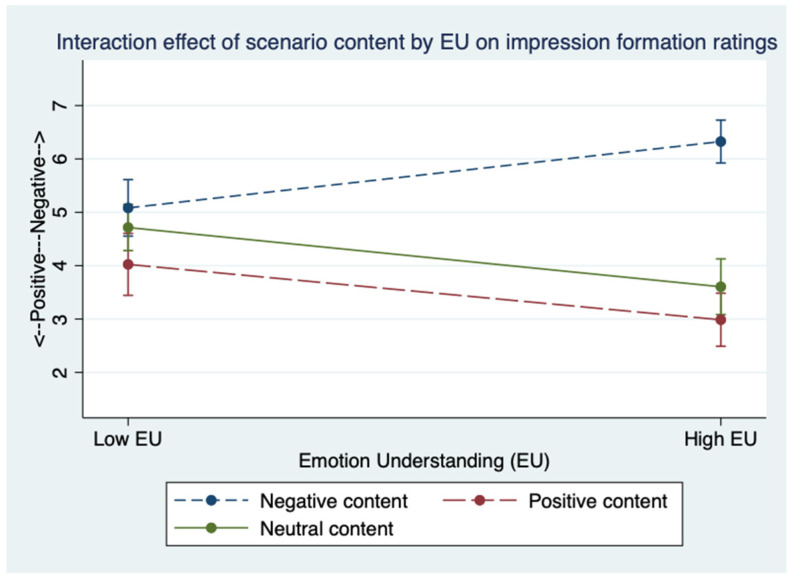
Interaction effect of EU (Emotion Understanding) by scenario content on ratings of Donald. High and low Emotion Understanding are calculated as ±1 SD from the mean.

Importantly, the results showed a significant interaction of scenario content by Emotion Understanding (Figure 2). Visual inspection shows that impression formation did not differ much across the three scenarios for low Emotion Understanding individuals, whereas for high Emotion Understanding individuals negative scenario showed a different pattern from neutral and positive scenarios. 

To test hypotheses, we calculated three simple slopes for the 3 vignette contents. The slope of Emotion Understanding in the negative scenario condition was positive and significant, *B* = 2.96, *p* < .001, LLCI = 1.55, ULCI = 4.37, indicating that as Emotion Understanding increased from low to high, negative ratings increased linearly. These results confirm Hypothesis 1. The slope of Emotion Understanding in the positive scenario was negative and significant, *B* = −2.47, *p* = 0.003, LLCI = −4.09, ULCI = −0.85, indicating that as it increased from low to high, impression formation ratings decreased linearly becoming more positive. Hence, Hypothesis 2 was confirmed. 

In the neutral scenario condition, the slope of Emotion Understanding was significant, *B* = −2.64, *p* = 0.003, LLCI = −4.38, ULCI = −0.91, with high Emotion Understanding participants giving more positive ratings to Donald than low Emotion Understanding participants. Thus, somewhat unexpectedly, in the neutral scenario, high Emotion Understanding individuals perceived more positive characteristics of Donald than did low Emotion Understanding individuals, even though the text did not include any explicit positive information about him. 

### 2.3. Moderated Mediation Analysis

We employed the Process Macro for SPSS (Hayes and Preacher 2014) to test Hypotheses 3 and 4 using a bootstrapping procedure with 20,000 bootstrapping samples to construct percentile bootstrap confidence intervals. This procedure can be employed to address power issues (Schoemann et al. 2017). All the variables were standardized before conducting analyses. For the negative scenario (Table 3), the test of *Path a* of the moderated mediation (Figure 1) revealed that high Emotion Understanding individuals retrieved more negative information than low Emotion Understanding individuals in the negative scenario, *β* = .22, *p* < .000, LLCI = .11 ULCI = .34. The test of the *Path b* showed that individuals higher in Emotion Understanding were more significantly influenced by the amount of retrieved negative information in rating negatively the vignette character, Donald, than individuals low in Emotion Understanding, *β* = .17, *p* = .02, LLCI = .02 ULCI = .32. The conditional indirect effect of negative scenario content on impression formation through retrieval of negative information was significant for average and high (+1 SD), but not for low Emotion Understanding individuals (−1 SD) (see Table 3), overall confirming Hypothesis 3.

For the positive scenario (Table 4), *Path a* was not significant. Meanwhile, *Path b* shows that individuals higher in Emotion Understanding were more significantly influenced by the amount of positive information recalled in rating positively Donald than low Emotion Understanding individuals, *β* = −.31, *p* < .01, LLCI = −.52 ULCI = −.10. Confirming Hypothesis 4, the conditional indirect effect of positive scenario on impression formation ratings through retrieval of positive information was significant for high (+1 SD) and average Emotion Understanding, but not for low Emotion Understanding (−1 SD) EI (see Table 4c).

## 3. Discussion

We tested the hypersensitivity hypothesis that high EI individuals, operationalized as individuals scoring high on the Emotion Understanding component of ability EI on the STEU, give greater weight to affectively valenced information than do low EI individuals. Results confirmed this prediction in that positive information about the target, Donald, increased high EI participants’ positive impressions more than it increased low EI participants’ positive impressions, and negative information increased their negative impressions more. In other words, evaluative information about Donald amplified the affective ratings of him in a direction consistent with the valence of that information, and more so for high than for the low EI participants. 

The unanticipated finding that high EI participants provided more positive ratings than low EI participants in the neutral condition, although not part of our hypotheses, cries out for an explanation. One interesting possibility is that this is a reflection of differences in positivity offset (e.g., Cacioppo et al. 1997; Ito and Cacioppo 2005) wherein, at low levels of evaluative activation, positive affect (i.e., approach motivation) is stronger than negative affect (i.e., withdrawal motivation)—an operating characteristic of the affect system that “fosters social cohesion” (Cacioppo et al. 2012, p. 55). It is interesting to note that the positive evaluation of Donald provided by high EI individuals in the neutral condition did not differ significantly from the positive evaluation provided by high EI individuals in the positive condition, *F* (1, 113) = 1.93, *p* > .05, which suggests that EI might in fact be a moderator of the positivity offset, with high EI individuals seeing neutral situations as somewhat more positive than do most individuals. This interpretation of the results is compatible with the idea of EI functioning as a sort of lens that alters the perception of reality in a way that amplifies its affective significance. 

Although we did not include any explicit positive valence information in the neutral scenario, a different explanation is that the context of the vignette—that of two friends hanging out together—might have primed concepts such as friendship with the result that the vignette might have had an unanticipated slightly positive overall tone, which in turn led high EI individuals to rate Donald more positively. Yet another possibility is that the positive ratings of Donald in the neutral condition might be related to the prosocial orientation that individuals high in EI, especially in emotion recognition, show in different contexts. It has been suggested (Schlegel 2020) that individuals high in emotion recognition might have a sort of implicit motivation to “be nice” to others and in this way avoid potential negative emotions derived from perceiving unpleasant emotions in others. 

Finally, the fact that participants recalled valenced idea units after reading the supposedly neutral version suggests that the neutral scenario was not fully neutral. We did not have the scenarios independently rated for valence, which might have helped to rule out this possibility, although it should be noted that the scenarios were intentionally constructed to be somewhat ambiguous with respect to their content to enable us to capture more variability in impression formation ratings.

Regarding the moderated mediation analysis, results while stronger for negative than for positive information, nevertheless substantially confirmed that EI affected the amount of recalled information, thereby intensifying valence-congruent affective ratings of Donald. Thus, overall, our results support the idea that EI may function as a magnifier that affects the cognitive processes involved in perceiving and understanding emotion-relevant information, as well as those involved in the retrieval of such information, at least when it is used as a basis for social judgment. 

Our results supporting the idea that high EI individuals are hypersensitive to emotions and emotion information are consistent with emerging findings regarding undesirable side effects of EI (Davis and Nichols 2016). In particular, hypersensitivity effects compatible with those we have reported here have been observed in studies related to stress management and responsivity discussed earlier (Baker et al. 2013; Bechtoldt and Schneider 2016; Matthews et al. 2006; Mikolajczak et al. 2009), suggesting that the hyper-awareness of high EI individuals may under certain circumstances contribute to ill health (Ciarrochi et al. 2002). 

Although our results suggest that amplificatory effects of negative information associated with high EI can exacerbate negative social judgments, they also show potentially beneficial effects of the amplification of positive information. In particular, individuals high in EI who read the positive scenario got a much more positive impression of Donald than low EI individuals. This result might explain why EI is often associated with quality of social interactions (e.g., Lopes et al. 2004). 

## 4. Limitations and Future Directions

Although we believe we have provided evidence in support of the conceptualization of EI as a magnifier of emotional experience, the study we presented has certain limitations. First, the study we have reported is but an initial effort to find direct and explicit support for our hypersensitivity hypothesis. As always, replications and different experimental paradigms would strengthen the case because it remains to be determined that other individual difference variables such as verbal fluency and intelligence were not contributing to the observed effects. However, this problem is to some extent mitigated by the fact that we found no evidence of superior recall of non-affective information by high EI individuals. 

Second, we observed the phenomenon of hypersensitivity with respect to only one component of ability EI, namely Emotion Understanding. However, we do not know whether amplification of perception and enhanced retrieval of emotional information might also be associated with other EI facets. Similar effects to the ones found in our study have been reported for the emotion perception branch of EI, which suggests that hypersensitivity may involve at least these two components of ability EI. We chose Emotion Understanding because it was the most pertinent given that our experimental task was essentially about understanding affective information presented in a text; hypersensitivity in other EI branches might emerge using different experimental paradigms, such as attentional processes as related to the EI component of emotion management, or fine-grained apperception of emotion information as related to emotion perception. Future research should shed light on the association between hypersensitivity and the different EI components. 

Third, although the current study provides support for the hypothesized functioning of EI as a magnifier of emotional experience, it does not address the question of whether and under what circumstances hypersensitivity might be beneficial or detrimental to thinking and behavior. It is important to note that we are not claiming that hypersensitivity to emotion and emotion information is by itself harmful. Individuals allocating more attention to emotional as opposed to neutral cues are faster and more accurate in recognizing emotions (Matthews et al. 2014). We believe that an effective way to think through how hypersensitivity might be related to beneficial vs. detrimental consequences is to think in terms of a 2 × 2 matrix in which amplification of positive and negative emotion information is crossed with outcome for the individual (advantageous vs. adverse). Although admittedly speculative, one might conjecture that both types of amplification could have beneficial and detrimental consequences for the individual. For example, the amplification of negative information might in some contexts lead people to be (appropriately) more cautious and vigilant, while in other contexts leading them to be unrealistically pessimistic. Amplification of positive information might in some context make people more careless or gullible, and in other contexts make them more realistically optimistic.

Hence, like other individual differences, hypersensitivity to emotions and emotion information associated with EI may help or hinder depending on the context. In addition to contextual considerations, another factor that might affect whether the magnifying effect may lead to positive or negative consequences is the overall availability of resources: when time and cognitive resources (such as attention) are lacking, hypersensitivity may lead to adverse effects if not balanced by additional regulatory processes. For example, detrimental effects of Emotion Understanding or Emotion Perception might emerge when paired with maladaptive, as opposed to adaptive, emotion regulation strategies.

Another potential explanation of how the hypersensitivity of high EI individuals might be linked to advantageous or adverse consequences that merits investigation is the question of whether there is an optimal level of EI beyond which it becomes counterproductive. For example, an interesting study analyzing the curvilinear effect of general intelligence on perceived leadership behavior (Antonakis et al. 2017) shows that the perception of leadership quality peaks at about 1.2 stand deviations above the mean IQ of the group members, after which it decreases. The effect of EI might follow a similar inverted U-shape trend, with positive effects occurring up to a certain level, and then detrimental effects starting to show at very high EI scores.

Further research is also needed to ascertain whether hypersensitivity is only associated with ability EI or whether it is also associated with trait EI. Studies reporting adverse effects of EI have been found in the trait EI literature. However, whether the reasons for such effects reside in the same type of processes that we have highlighted remains to be determined. Research has shown that ability and trait EI may predict the same outcomes, but through different paths (e.g., Udayar et al. 2020). Ability and trait EI might rely on different ways of processing emotion information, so that, for example, individuals high in trait EI might process positively valenced information differently than individuals high in ability EI; for example, the former might privilege valence over accuracy of emotion detection. A replication of our findings with trait EI measures is therefore warranted. 

In conclusion, we have provided initial support for the hypersensitivity hypothesis and the associated idea that EI functions as a magnifier of emotional experience, making individuals high in EI particularly sensitive to affective information. Such individuals are hypersensitive in the sense that they feel more intense emotions, have a more fine-grained apperception of affective responses in themselves and others, have a strong sensitivity to the meaning and effects of emotions, and have more reactive attentional mechanisms associated with emotion information. Much research is needed to fully explore the implications of this conceptualization of EI and for the question of what it really means to be emotionally intelligent.

## Figures and Tables

**Table 1:** Mean, standard deviation, and correlation of the Study variables.

	Mean	St. Dev	1	2	3	4	5	6	7	8	9	10	11	12	13
1. Age	29.45	9.73	1.00												
2. Sex (F = 2)	1.27	.44	.19	1.00											
3. Negative scenario	.35	.48	−.00	−.05	1.00										
4. Positive scenario	.36	.48	.02	.01	−.56 **	1.00									
5. Neutral scenario	.28	.45	−.01	.04	−.47 **	−.48 **	1.00								
6. Mood_overall	15.61	3.52	.00	.00	−.06	.01	.06	1.00							
7. EmoUnderstanding (EU)	.49	.21	.32	.14	.05	.07	−.13	−.04	1.00						
8 IF_Negative	4.79	1.96	−.09	−.05	.56 **	−.44 **	−.13	−.13	−.18	1.00					
9. IF_Positive	5.70	1.83	−.01	.10	−.57 **	.47 **	.09	.09	−.15	−.54 **	1.00				
10. IF_overall	4.56	1.70	−.06	−.09	.64 **	−.52 **	−.13	−.12	−.05	.91 **	−.84 **	1.00			
11. Positive retrieval	.75	1.40	−.03	−.02	−.30 **	.53 **	−.25 **	.02	−.12	−.17	.35 **	−.28 **	1.00		
12. Negative retrieval	1.03	1.73	.02	.03	.68 **	−.36 **	−.34 **	−.09	.24 **	.52 **	−.69 **	.67 **	−.27 **	1.00	
13. Neutral retrieval	4.05	2.52	.18	−.05	−.31 **	−.01	.34 **	.09	.10	−.33 **	.33 **	−.38 **	−.23 **	−.42 **	1.00

*Note*. EmoUnderstanding = Emotion Understanding, IF_Negative = Impression Formation ratings with negative adjectives, IF_Positive = Impression Formation ratings with positive adjectives, IF_overall = Impression Formation ratings with negative and positive (reversed). * *p* < .05, ** *p* < .01.

**Table 2:** Regression results of scenario content, emotion understanding, and control variables on impression formation.

	Unstand.	Standardized	Robust				
	Coef.	Coef.	Std. Err.	t	P>t	[95% Conf.	Interval]
Mood_overall	−.01	−.02	.04	−.33	.74	−.08	.06
Age	−.01	−.04	.01	−.55	.59	−.03	.02
Sex	−.18	−.05	.24	−.76	.45	−.66	.29
Positive scenario	.24	.07	.60	.41	.68	−.95	1.43
Neutral scenario	.98	.27	.54	1.80	.08	−.10	2.06
Emotion Understanding (EU)	2.96	.37	.71	4.18	.00	1.55	4.37
EUXpositive scenario	−5.43	−.88	1.05	−5.17	.00	−7.51	−3.35
EUXneutral scenario	−5.60	−.77	1.07	−5.21	.00	−7.74	−3.47

**Table:** **(a) Outcome: negative retrieval (R^2^ = .5**8).

	Stand. coeff.	se	t	p	LLCI	ULCI
Negative scenario	.60	.06	9.58	.00	.47	.72
Emotion Underst. (EU)	.24	.06	3.72	.00	.11	.36
ScenarioXEU	.22	.06	3.80	.00	.11	.34
Mood_overall	.06	.07	.91	.36	−.07	.19
Sex	.03	.06	.49	.62	−.09	.15
Age	−.09	.06	−1.38	.17	−.22	.04

**Table:** **(b) Outcome: impression formation (R^2^ = .5**8).

	Stand. coeff.	se	t	p	LLCI	ULCI
Negative scenario	.30	.09	3.29	.00	.12	.47
Negative retrieval	.47	.11	4.32	.00	.26	.69
Emotion Underst. (EU)	−.18	.07	−2.47	.02	−.33	−.04
ScenarioXEU	.17	.07	2.27	.02	.02	.32
Mood_overall	−.07	.07	−.99	.32	−.21	.07
Sex	−.07	.07	−.99	.32	−.20	.07
Age	.00	.07	−.01	.99	−.14	.14

**Table:** **(c) Results of the indirect effect: Negative scenario → Negative retrieval → Impression formation at different levels of emotion** **understanding.**

Emotion Underst.	Effect	BootSE	BootLLCI	BootULCI
.97	.12	.07	−.02	.24
.04	.29	.08	.12	.45
1.06	.54	.11	.33	.78

**Table:** **(a) Outcome: positive retrieval (R^2^ = .3**2).

	Stand. coeff.	se	t	p	LLCI	ULCI
Positive scenario	.49	.07	6.70	.00	.34	.63
Emotion Underst. (EU)	−.15	.08	−1.90	.06	−.30	.01
ScenarioXEU	−.03	.08	−.43	.67	−.18	.12
Mood_overall	.05	.08	.67	.50	−.10	.21
Sex	.04	.08	.47	.64	−.11	.18
Age	−.01	.08	−.17	.87	−.17	.14

**Table:** **(b) Outcome: impression formation (R^2^ = .3**3).

	Stand. coeff.	se	t	p	LLCI	ULCI
Positive scenario	−.40	.10	−4.03	.00	−.59	−.20
Positive retrieval	−.21	.11	−1.84	.07	−.44	.02
Emotion Underst. (EU)	−.01	.09	−.08	.94	−.18	.17
ScenarioXEU	−.31	.11	−2.92	.00	−.52	−.10
Mood_overall	−.08	.09	−.96	.34	−.26	.09
Sex	−.07	.08	−.85	.40	−.23	.09
Age	−.06	.09	−.67	.50	−.23	.11

Note: LLCI 95% lower-limit confidence interval, ULCI 95% upper-limit confidence interval.

**Table:** **(c) Results of the indirect effect: Positive scenario → Positive retrieval → Impression formation at different levels of emotion** **understanding.**

Emotion Underst.	Effect	BootSE	BootLLCI	BootULCI
.97	.05	.06	−.05	.19
.04	−.11	.05	−.23	−.01
1.06	−.24	.09	−.44	−.09

## Data Availability

The data presented in this study are available on request from the corresponding author.

## Appendix A. Vignettes Employed in the Study. The Valenced Sentences Are Highlighted

### Appendix A.1. Negative Content

I ran into my old acquaintance Donald the other day and I decided to go over and visit him, since by coincidence we took our vacations at the same time. Soon after I arrived, a salesman knocked at the door, but Donald refused to let him enter. He shouted that by no means he wanted to waste his time talking to a salesman. He also told me that he was refusing to pay his rent until the landlord repaints his apartment and that he was thinking to file a lawsuit against the landlord because he did not have a regular lease contract. We talked for a while, had lunch, and then went out for a ride. We used my car, since Donald’s car had broken down that morning, and he told the garage mechanic that he would have to go somewhere else if he couldn’t fix his car that same day. We went to the park for about an hour and then stopped at a hardware store. Donald bought some small gadget, and then I heard him demand his money back from the sales clerk. I couldn’t find what I was looking for, so we left and walked a few blocks to another store. The Red Cross had set up a stand by the door and asked us to donate blood. Donald lied by saying he had diabetes and therefore could not give blood. I hadn’t noticed it before, but when we got to the store, we found that it had gone out of business. It was getting kind of late, so I took Donald to pick up his car and we agreed to meet again soon.

### Appendix A.2. Positive Content

I ran into my old acquaintance Donald the other day and I decided to go over and visit him, since by coincidence we took our vacations at the same time. Soon after I arrived, a salesman knocked at the door, and Donald spent some time kindly talking to him. He did not buy anything and he explained to the salesman that he had just received a visit and wished to go out with his friend as soon as possible. Donald lived in a nice apartment and he told me he was grateful that the landlord had just agreed to repaint it. We talked for a while, had lunch, and then went out for a ride. We used my car, since Donald’s car had broken down that morning, and he told the garage mechanic that he was available to pay extra money in order to have the car fixed the same day. We went to the park for about an hour and then stopped at a hardware store. Donald bought some small gadget and while he was waiting for me he started to make funny jokes with the sales clerk. I couldn’t find what I was looking for, so we left and walked a few blocks to another store. The Red Cross had set up a stand by the door and asked us to donate blood. Donald insisted to stop over and give blood. I hadn’t noticed it before, but when we got to the store, we found that it had gone out of business. It was getting kind of late, so I took Donald to pick up his car and we agreed to meet again soon.

### Appendix A.3. Neutral Content

I ran into my old acquaintance Donald the other day and I decided to go over and visit him, since by coincidence we took our vacations at the same time. Donald’s apartment was located about a mile from the city center; I got there by car and was lucky to easily find a spot for parking right in front of his place. When I arrived, Donald showed me his apartment: he lived on the second floor of a new building in a one-bedroom flat with a large living-room and a balcony. We talked for a while about what we had been doing lately, then we had lunch, and finally went out for a ride. We used my car, since Donald’s car had broken down that morning, and he asked the garage mechanic to have the car fixed the same day. We had a walk in the park for about an hour and then we stopped at a hardware store. Donald bought some small gadget including some paint for repainting his apartment; I couldn’t find what I was looking for, so we left and walked a few blocks to another store. On the way to the store we saw The Red Cross that had set up a stand and was looking for volunteers to donate blood. I hadn’t noticed it before, but when we got to the store, we found that it had gone out of business. A notice informed the hardware store would be replaced by a grocery store with organic food that would open in two months. It was getting kind of late, so I took Donald to pick up his car and we agreed to meet again soon.
